# Coded and uncoded chronic kidney disease: an explainer

**DOI:** 10.3399/BJGP.2025.0228

**Published:** 2025-08-25

**Authors:** Stuart Stewart, Philip A Kalra, Evangelos Kontopantelis, Tom Blakeman, Smeeta Sinha

**Affiliations:** 1 University of Manchester and the Donal O’Donoghue Renal Research Centre, Northern Care Alliance NHS Foundation Trust, Manchester, UK

## Introduction

To clinicians and those familiar with clinical coding, the terms coded and uncoded chronic kidney disease (CKD) may appear self-evident — a disease with or without a diagnostic clinical code in the electronic health record (EHR). However, for others these terms are ambiguous or unknown. Is coded CKD the same as diagnosed CKD? Is uncoded CKD the same as undetected CKD? Such confusion even occurs during peer review of CKD studies.^1^ These terms are simple to the eye but laden with complexity that leaves room for misinterpretation and misunderstanding.

The implications of these distinctions are far reaching. Research shows lower risk of acute kidney injury (AKI), unscheduled care, and even death for patients with coded versus uncoded CKD^2,3^ owing to the multiple protective effects afforded by recording diagnostic clinical codes in a patient’s EHR.^4^ However, these health protective effects are under-recognised in UK primary care, as evidenced by large numbers of patients with uncoded CKD stages 3–5.^4^ With research increasingly examining the impact of coding status in CKD^5–9^ it is necessary to clarify and expand upon the meaning of these increasingly used terms.

## Terminology

**Figure 1.**
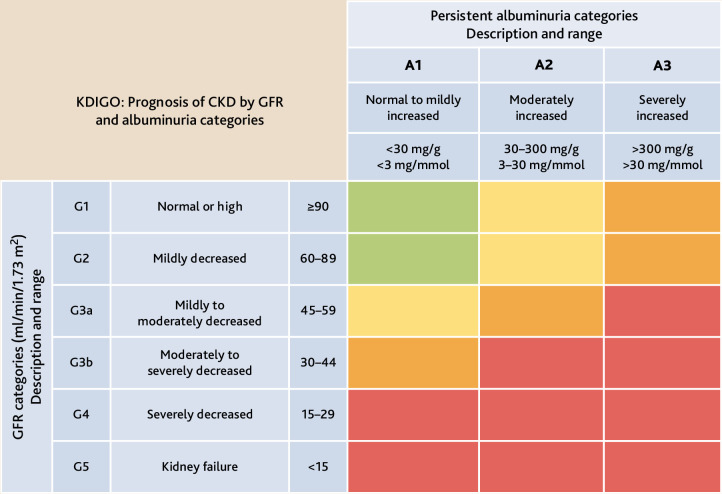
KDIGO chronic kidney disease nomenclature. Used with permission of KDIGO. Green = low risk (if no other markers of kidney disease, no CKD); yellow = moderately increased risk; orange = high risk; red = very high risk. CKD = chronic kidney disease. GFR = glomerular filtration rate. KDIGO = Kidney Disease: Improving Global Outcomes.

The terms *coded* and *uncoded CKD* reflect not only the presence of a disease — as defined by the Kidney Disease: Improving Global Outcomes (KDIGO) classification of CKD (Figure 1)^10^ — but also its formal documentation in the EHR through the process of clinical coding. Table 1 summarises what coded and uncoded CKD does and does not mean.

**Table 1.** Descriptive summary of coded and uncoded CKD

	What it is	What it is not
**Coded CKD**	The formal assignment of a diagnostic clinical code (for example, SNOMED CT, ICD-10, retired Read codes) for patients who meet the KDIGO diagnostic criteria^10^ for CKD. Generally, coded CKD represents patients with confirmed and diagnosed CKD who have been coded appropriately. Occasionally, it may capture a cohort of patients with a CKD code irrespective of whether diagnostic criteria are met, a potential source of misclassification.	Coded CKD does not guarantee diagnostic accuracy, patient disclosure or understanding, or active disease management. It does not reflect disease severity, or which diagnostic criteria were met. It is not synonymous with active or progressive disease.
**Uncoded CKD**	Patients meeting KDIGO diagnostic criteria for CKD^10^ without a corresponding diagnostic code in the EHR. The absence of coding reflects the absence of the behaviour of coding, not the absence of disease. It may also be described as patients with biochemical evidence of CKD without a diagnostic code.^4^ Patients with perpetually uncoded CKD during 1) their lifetime or 2) a study period may be described as *never-coded CKD*.	While a patient with uncoded CKD may have undiagnosed CKD, uncoded is not synonymous with undiagnosed. Uncoded CKD doesn’t mean undetected CKD as CKD is detected at the point of laboratory testing in line with KDIGO diagnostic criteria.^10^ It doesn’t mean a lack of awareness by the patient or clinician, although this can occur.^4^ It doesn’t mean a patient’s condition or care has been overlooked; there may be valid reasons for not coding a diagnosis such as in patients who are terminally ill.^4^ Uncoded CKD is not a static or homogeneous entity, nor a simple binary opposite of coded CKD — it is a dynamic state with a time component where most patients eventually receive a diagnostic code.^4^

EHR = electronic health record. ICD-10 = International Classification of Diseases, 10th revision. KDIGO = Kidney Disease: Improving Global Outcomes. SNOMED CT = Systematised Nomenclature of Medicine Clinical Terms.

## Implications for practice, policy, and research

Refining and expanding the definitions of coded and uncoded CKD has implications for clinical practice, policy, and health research*.^10^* Our key recommendations to improve coding of CKD in the EHR are described in Table 2 *.*

**Table 2.** Recommendations to improve recording of CKD in the electronic health record

Clinicians across both primary and secondary care have a responsibility for diagnosis and coding of CKD in the EHR.^1,21^ Diagnosis of early CKD (stages 1 and 2) is dependent on albuminuria testing (or the presence of other markers of kidney damage).^22^ In order to improve detection, diagnosis, and coding of early CKD, clinicians and policymakers must review barriers to and enablers of albuminuria testing.^4^ Albuminuria testing should be used in patients at high risk of CKD.^22^ Clinicians can readily identify patients with potentially uncoded CKD in modern EHR systems by searching for patients with 2 × eGFR measurements <60 mL/min/1.73m^2^ at least 90 days apart without a diagnostic clinical code or persistent albuminuria.^4^ In England, clinicians can use the CVDPREVENT tool^23^ to quantify the number and proportion of uncoded and coded CKD cases at a practice, local, and regional level. Such tools integrate into established quality improvement activity workflows, which could be conducted at intervals accordant with local prevalence of CKD.^4^

CKD = chronic kidney disease. eGFR = estimated glomerular filtration rate. EHR = electronic health record.

### Clinical practice

#### Patient-level management

Evidence shows coded CKD is associated with lower risk of AKI,^3^ unscheduled care,^3^ cardiovascular admissions,^7^ heart failure admissions,^7^ and even death.^3^ These benefits are a consequence of the downstream effects of clinical coding: automated prescribing alerts to avoid nephrotoxic medications and encourage protective medications (for example, renin–angiotensin system inhibitors, SGLT2 inhibitors, lipid-lowering therapies); increased opportunities for evidence-based care (for example, pharmacological recommendations according to albuminuria, monitoring frequency according to CKD stage); and vaccination reminders. Given the prevalence of CKD in the context of multiple long-term conditions, coding CKD offers unique benefits through the automation of cross-disease management alerts (for example, blood pressure recommendations for pre-existing hypertension).^4^ Moreover, coding helps clinicians automate a series of high-yield clinical tasks that are essential for managing individual patient’s health and population health at scale.^4^

Where clinicians may hesitate to code CKD in patients with advanced illnesses, coding a diagnosis of CKD still creates opportunities for helpful automated prescribing alerts for drugs with increased risk of AKI,^11^ side effects, or toxicity in the context of kidney impairment.^4^ When treating patients with coded CKD, clinicians should review patient awareness and understanding of the diagnosis and offer at least an annual review for CKD.^4^

#### Population-level management

Clinical coding at the patient level is what enables proactive CKD care to happen at scale, and is therefore a core tool for clinicians to identify and recall patients for chronic disease reviews.^6^ However, given the prevalence of uncoded CKD, identifying uncoded cases is essential, and can easily be done in modern EHRs by searching for patients based on eGFR and/or albuminuria results.^4–6^ As such, effective quality improvement (QI) programmes are available for UK healthcare professionals to tackle the complexity of managing CKD both at patient and population levels.^12^ In the UK, clinicians can refer to guides on how to code CKD in the EHR.^6^ Research demonstrates the efficacy of QI programmes to improve coding in CKD.^5,7^ The burden of uncoded CKD in secondary care has been identified as a health system problem that requires a populationlevel approach.^1^

### Research

#### Dynamic coding status

Most patients with uncoded CKD eventually become coded, making it difficult to isolate the nuanced effects of being uncoded on health outcomes. Considering the temporal dimension in time-to-event analyses should be prioritised where possible. Patients who meet diagnostic criteria for CKD but are perpetually uncoded may be described as ‘never-coded CKD’ — a subset of uncoded CKD. This group of patients may reflect outliers or extreme cases, therefore caution is recommended when comparing this group with patients with coded CKD, even in a matched setting.

#### Coding status as a research topic

Clarifying the definitions of coded and uncoded CKD contributes to the wider body of research investigating the impact of uncoded disease states (for example, pre/diabetes,^13,14^ hypertension^15^ on health outcomes). Furthermore, it contributes to the ongoing discourse around automated coding of disease states within the EHR and their potential for improving care and health outcomes.^16^ Consideration of the ethical implications of automated clinical coding is necessary, as illustrated through the use of automated frailty scores to potentially ration finite critical care resources during the COVID-19 pandemic.^17^

### Policy

#### Data quality

High-quality health research data are discussed as central to addressing gaps in kidney disease care (health inequity) and health outcomes (health inequality).^10^ At the most basic level, this starts with accurate recording of CKD using clinical codes in EHRs so that policymakers can design policies that are underpinned by reliable data.

#### Early detection

While population screening for CKD is not endorsed by the national screening committee, targeted case finding among at-risk groups is,^4^ with the aim of earlier detection of CKD. Less than 25% of patients admitted to hospital experiencing AKI were screened for CKD, within 90 days of discharge.^18^ Patients with early stages of CKD are less likely to be coded than later stages.^2^ These findings illustrate missed opportunities for timely detection, diagnosis, and coding of early CKD. If clinicians are to intervene earlier to slow disease progression and reduce risk of adverse health outcomes, barriers to screening at-risk groups (and therefore detection of early stages of CKD through albuminuria testing) and barriers to clinical coding of early stages must be addressed.^4^. In England, a new strategy for the NHS that supports a shift from treatment towards prevention offers opportunities to achieve ambitions of early detection.^19^ Such a wholesale change in clinical practice requires policy changes that incentivise early detection through albuminuria testing.^20^ Should earlier stages of CKD be more accurately coded, sharing of this data with the UK renal registry could enable us to better understand disease progression from an earlier stage.

#### Quality of care

While the risk of adverse health outcomes can be lowered by coding patients with uncoded CKD,^5^ coding the diagnosis does not guarantee disclosure of the diagnosis or quality of care.^4^ Variations in quality of care are observed since the loss of financially incentivised CKD care in English primary care in 2015.^4^ As such, policy must address both the major burden of *uncoded CKD*, and re-examine how funding can be strategically used to improve quality of care for all patients with *CKD* — coded and uncoded.^4^

#### Conclusions

Specifically, this commentary addresses a gap in the language of coding in CKD. However, more broadly, this commentary contributes to the evolving discourse on the interplay between clinician behaviour, clinical coding as a digital health intervention, and the impact on patient outcomes and health inequalities. Clinical coding is an essential tool in the management of patient and population health. As EHRs offer fertile ground to explore CKD and other chronic disease care, precise and comprehensive terminologies are essential for conducting rigorous and reproducible research.
